# The Antitumor Natural Compound Falcarindiol Disrupts Neural Stem Cell Homeostasis by Suppressing Notch Pathway

**DOI:** 10.3390/ijms19113432

**Published:** 2018-11-01

**Authors:** Tae-Jun Kim, Hyun-Sook Kwon, Mingyu Kang, Hyun Hee Leem, Kyung-Ha Lee, Do-Yeon Kim

**Affiliations:** 1 Department of Pharmacology, School of Dentistry, Kyungpook National University, Daegu 41940, Korea; toy5988@naver.com (T.-J.K.); alsrb5788@naver.com (M.K.); 2 National Development Institute of Korean Medicine, Gyeongsan, Gyeongsangbuk-do 38540, Korea; sook4269@naver.com (H.-S.K.); ms_hee@naver.com (H.H.L.); 3 Department of Cosmetic Science and Technology, College of Bio-industry, Daegu Haany University, Gyeongsan 38610, Korea; 4 Department of Pharmacology, School of Dentistry, Brain Science and Engineering Institute, Kyungpook National University, Daegu 41940, Korea

**Keywords:** falcarindiol, neural stem cell, Notch pathway, apoptosis, differentiation

## Abstract

Neural stem cells (NSCs) are undifferentiated, multi-potent cells that can give rise to functional neurons and glial cells. The disruption in NSC homeostasis and/or the impaired neurogenesis lead to diverse neurological diseases, including depression, dementia, and neurodegenerative disorders. Falcarindiol (FAD) is a polyacetylene found in many plants, and FAD shows the cytotoxicity against breast cancers and colon cancers. However, there is no research on the consequence of FAD treatment in normal stem cells. Here, we suggest that FAD has anticancer roles against glioblastoma cells by inducing the differentiation of glioblastoma stem-like cells, as well as activating apoptosis pathway in glioblastoma cells. On the other hand, we also show that FAD has detrimental effects by disrupting the maintenance of normal NSCs and altering the balance between self-renewal and differentiation of NSCs.

## 1. Introduction

Neural stem cells (NSCs) are undifferentiated, multi-potent cells present in the developing and adult nervous systems. NSCs are capable of self-renewal and have long-term proliferative potential [1]. Differentiation of NSCs into neurons, astrocytes, and oligodendrocytes is controlled precisely, and the balance between self-renewal and differentiation in NSCs is critical for the development of the central nervous system [2]. Although some studies insisted that new neurons are robustly generated only in embryos and early postnatal life [3,4], accumulating evidence has shown that the neurogenesis in adult human brain persists throughout aging [5]. These newborn neurons contribute to the structural and functional integrity of the adult brain and the persistence of cognitive function throughout life [6]. The disruption in NSC homeostasis and/or the impaired neurogenesis derived from NSC are highly associated with several neurological diseases, including depression, dementia, and Alzheimer’s disease [7]. In addition, it was reported that NSCs in hypothalamus control ageing speed in mammals [8], highlighting the importance of NSCs in the maintenance of the nervous system.

Falcarindiol (FAD) is a polyacetylene found in many dietary plants, and it has been shown to have a variety of physiological activities, such as anti-inflammation, antibacterial, and hepatotoxicity suppression [9,10,11]. In particular, anti-proliferative and antitumor functions of FAD were confirmed repeatedly, in colon cancer and breast cancer cells [12,13]. Unfortunately, however, there is little research on the consequence of FAD treatment in the fate determination of stem cells.

In this study, we show that FAD has cytotoxic effects on glioblastoma cells. Based on the previous reports that cancer stem cells have been regarded as the culprits of tumor recurrence, due to their high resistance to chemo- and radio-therapy [14], we evaluate the anticancer role of FAD targeting glioblastoma stem-like cells, as well as glioblastoma cells. However, we also give a warning that FAD could disrupt the maintenance of normal NSCs and FAD would alter the balance between self-renewal and differentiation in NSCs by inhibiting Notch pathway.

## 2. Results

### 2.1. FAD Shows the Antitumor Effect Against Glioblastoma Cells

**Figure 1:**
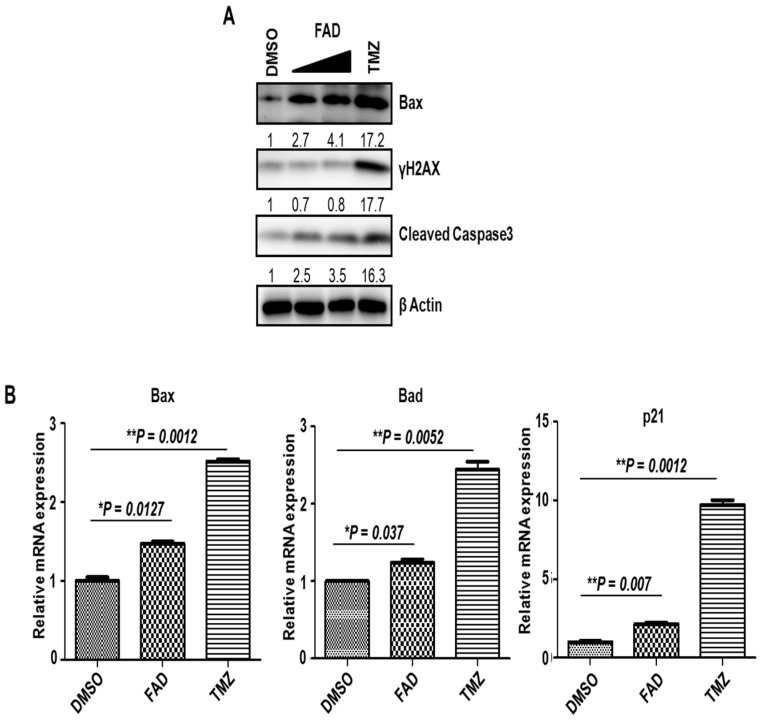
Cytotoxicity driven by falcarindiol (FAD) on glioblastoma cells. (**A**) Western blot analysis of Bax, γH2AX, and cleaved caspase 3 in U373 glioblastoma cells after treatment of dimethyl sulfoxide (DMSO), FAD (10 μM, 40 μM), and TMZ (200 μM) for 3 days. β Actin was used as a loading control. The relative band intensities of each proteins are shown below the bands. The intensities of vehicle treated lane were arbitrarily set as 1. (**B**) The mRNA expression of Bax, Bad, and p21 after treatment of DMSO, FAD (40 μM), and TMZ (200 μM) for 3 days in glioblastoma cells, as measured by real-time reverse transcription-polymerase chain reaction (RT-PCR) analysis. The mRNA level of DMSO-treated cells was set to 1. Representative results from multiple experiments are shown.

FAD was isolated with the bioactivity-guided fractionation of MeOH extract from *Saposhnikovia divaricate* that belongs to the Umbelliferae family, using column chromatography and thin layer chromatography (TLC) (Supplementary Figure S1A,B). To test whether FAD shows the cytotoxicity on glioblastoma cells, we treated U373 glioblastoma cells with two different doses (10 μM or 40 μM) of FAD. The cytotoxic effects were determined by the phosphorylation of H2AX (γH2AX), a biomarker of DNA damage, and the cleaved form of caspase3, a marker for apoptotic cell death, through immunoblot assay. Temozolomide (TMZ) [15,16], an oral alkylating agent used to treat glioblastoma multiforme (GBM) and astrocytomas, was used as a positive control. Compared to vehicle, FAD clearly induced apoptosis of glioblastoma cells at both concentrations we used (Figure 1A). Interestingly, however, FAD did not enhance γH2AX signals, indicating that FAD seems to act in a different manner on cells than TMZ. We also observed the increased expression of pro-apoptotic Bax gene by FAD treatment. To confirm the antitumor effect of FAD, we evaluated mRNA expressions of several pro-apoptotic genes (Figure 1B). The mRNA levels of pro-apoptotic members of the Bcl-2 gene family, Bax and Bad, were significantly upregulated by FAD treatment. Although p21 has been regarded as an apoptosis inhibitor rather than activator, p21 does not suppress apoptosis of cancers under nongenotoxic apoptotic signal [17]. Rather, recent evidence suggests that p21 has proapoptotic functions that supports our data [18,19]. Together, our results showed that FAD clearly has the anticancer effect on glioblastoma cells, although to a lesser extent than TMZ.

### 2.2. FAD Reduced the Stemness of Cancer Stem-Like Cells in Glioblastoma

**Figure 2:**
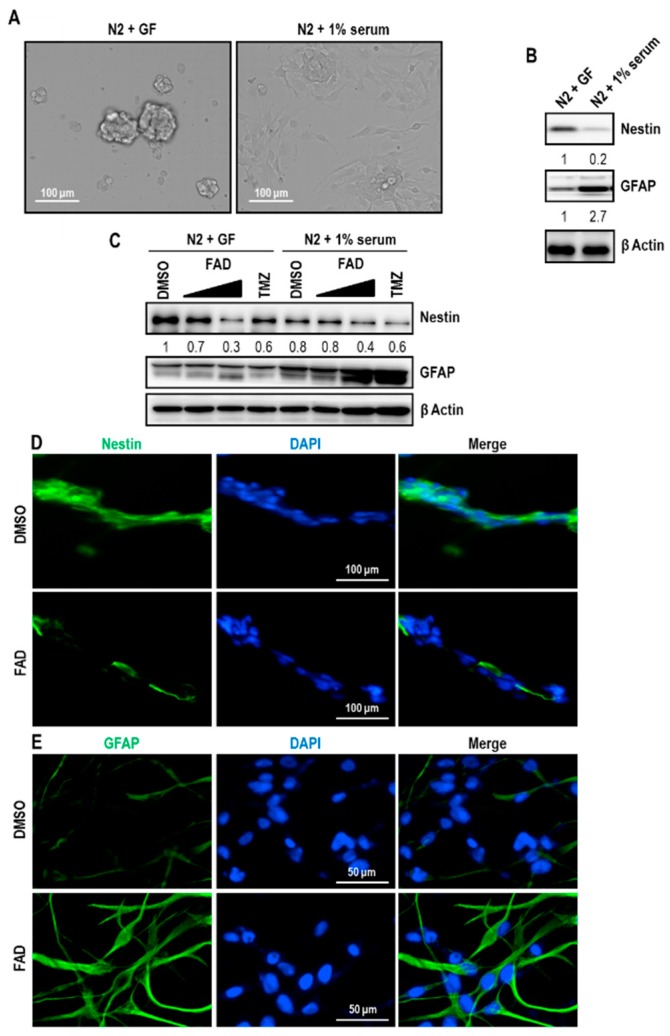
Downregulation of the stemness of glioblastoma stem-like cells by FAD treatment (**A**) Cell morphology of U373 glioblastoma cells in two different culture conditions. For the enrichment of glioblastoma stem-like cells, cells were maintained under N2 culture media supplemented with 20 ng/mL EGF and bFGF (N2+GF). Spheres are maintained with N2 media including 1% fetal bovine serum (FBS) for differentiation induction (N2 + 1% serum). (**B**) Western blot analysis of Nestin and GFAP in undifferentiated or differentiated glioblastoma cells. β Actin was used as a loading control. The relative band intensities of Nestin and GFAP proteins are shown below the bands. The intensities of N2+GF lane were arbitrarily set as 1. (**C**) Western blot analysis of Nestin and GFAP in undifferentiated or differentiated glioblastoma cells after treatment of DMSO, FAD (10 μM, 40 μM), and TMZ (200 μM) for 3 days. β Actin was used as a loading control. The relative band intensities of Nestin are shown below the bands. The intensities of vehicle treated lane were arbitrarily set as 1. (**D** and **E**) Immunofluorescence analysis of Nestin (**D**) and GFAP (**E**) expression in DMSO or FAD treated cells. Nuclear DAPI (4′,6-diamidino-2-phenylindole) staining is in blue. These are representative results of counting 10 random fields of view in each group.

The antitumor characteristics of FAD were already examined in other tumor types, including breast cancer and colorectal cancer [12,13]. However, the influence of FAD on cancer stem cells still remains unanswered. To this end, we enriched cancer stem-like cell populations in U373 glioblastoma by maintaining cells with serum-free culture media supplemented with 20 ng/mL epidermal growth factor (EGF) and basic fibroblast growth factor (bFGF). As earlier reported [20], these cells grew spherically without attached cells and were able to be propagated continuously (Figure 2A). When spheres were induced to differentiate with 1% serum containing media without growth factors, cells lost the sphere-forming ability and grew in monolayer. To verify the enrichment of cancer stem-like cells under sphere-forming conditions, we checked glioma stem cell marker by immunoblot assay. As expected, self-renewable U373 spheres highly expressed Nestin under serum-free condition (Figure 2B). When cells were enforced to differentiate, Nestin expression was dramatically downregulated. On the other hand, the level of glial fibrillary acidic protein (GFAP), a marker of differentiated astrocyte, was upregulated upon differentiation condition, suggesting that cancer stem-like cell populations in U373 glioblastoma have morphological and molecular characteristics of cancer stem cells.

To explore the effect of FAD on undifferentiated tumor initiating cells, we treated FAD on undifferentiated and differentiated glioblastoma cells. Interestingly, Nestin expression was dramatically reduced by 40 μM FAD treatment in serum-free condition (Figure 2C). In contrast, GFAP level was increased by high-dose FAD challenge, suggesting that FAD disrupts the homeostasis of glioblastoma stem-like cells and promotes differentiation. To confirm the inhibitory role of FAD in the maintenance of the stemness, we performed immunostaining. Consistent with the immunoblot data, the intensities of Nestin and GFAP signals were significantly diminished and enhanced, respectively, by FAD treatment (Figure 2D,E). Taken together, FAD shows the antitumor effect through (1) the induction of apoptotic pathway (Figure 1) and (2) the alteration of glioblastoma stem-like cell maintenance (Figure 2).

### 2.3. FAD Disrupts the Maintenance of Normal NSCs

**Figure 3:**
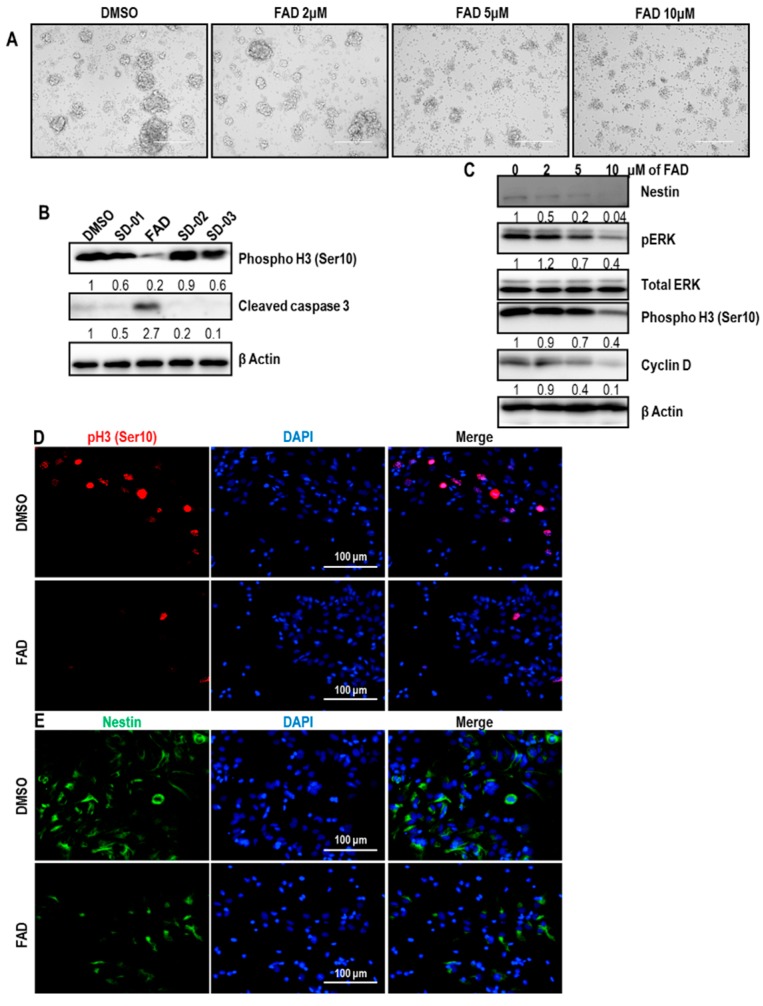
Disruption of the cellular maintenance by FAD in normal neural stem cells (NSC)s. (**A**) Sphere-forming capacity of NSCs in the absence (DMSO) or at various concentrations of FAD. Scale bar = 100 μm. (**B**) Western blot analysis of phosphor Histone 3 (Ser 10) and cleaved caspase 3 in normal NSCs after treatment of DMSO, FAD, and other single compounds (10 μM) for 3 days. β Actin was used as a loading control. The relative band intensities of each proteins are shown below the bands. The intensities of vehicle treated lane were arbitrarily set as 1. (**C**) Western blot analysis of Nestin, phosphor ERK, total ERK, phosphor Histone 3 (Ser 10), Cyclin D in DMSO or FAD-treated cells. Total ERK was used as a loading control for phosphor ERK. In other cases, β Actin was used as a loading control. The relative band intensities of each proteins are shown below the bands. The intensities of vehicle treated lane were arbitrarily set as 1. (**D**,**E**) Immunofluorescence analysis of phosphor Histone 3 at Ser 10 (shown in red) (**D**) and Nestin (shown in green) (**E**) expression in DMSO or FAD treated cells. Nuclear DAPI (4′,6-diamidino-2-phenylindole) staining is in blue. Cells were maintained under N2+GF media. These are representative results of counting 10 random fields of view in each group.

The cell fate control of glioblastoma stem-like cells by FAD raised a possibility that FAD could affect the proliferation or differentiation capability of normal NSCs. To test this hypothesis, we monitored the morphology of proliferating NSCs after treatment of vehicle or FAD. Compared to glioblastoma stem-like cells, NSCs lost the sphere-forming capacity when treated with much lower concentrations of FAD (Figure 3A). To examine the cytotoxic effect mediated by FAD on NSCs, we checked the level of the active form of caspase 3. Clearly, FAD induced the production of cleaved caspase 3, meaning that FAD promotes cell death of normal NSCs (Figure 3B). Because earlier reports demonstrated that FAD induced the autophagy [12,13], we analyzed the autophagy marker protein LC3. However, LC3-II/LC3-I ratio and p62 protein levels were not changed by FAD treatment, suggesting that the activation of apoptosis of NSCs would be autophagy-independent manner (Supplementary Figure S2). We also monitored the huge reduction of the mitotic marker, phosphor Histone 3 at Ser 10, after FAD challenge, suggesting that FAD disturbs the proliferating potential of NSCs (Figure 3B). On the other hand, when we treated NSCs with other compounds that were extracted from same source with FAD, the level of phosphor Histone 3 at Ser 10 remained unchanged and active caspase3 was not detected, confirming that the cytotoxic effect on NSCs is driven by FAD. Consistent with Figure 3B, phosphor ERK and Cyclin D levels were downregulated by FAD administration especially at the concentration of 10 μM (Figure 3C). Similar to glioblastoma stem-like cells, NSCs failed to express the stemness marker Nestin when treated with 10 μM of FAD. To confirm the suppressive role of FAD on the proliferation and maintenance of NSCs, we performed immunostaining. In line with the immunoblot results, the number of phosphor Histone 3 at Ser 10 positive cells were dramatically decreased by FAD treatment (Figure 3D). Also, FAD strongly reduced the expression of Nestin (Figure 3E). Collectively, these data suggest that FAD disrupts the stemness maintenance and proliferating potential of NSCs.

### 2.4. FAD Enhances the Differentiation of NSCs

**Figure 4:**
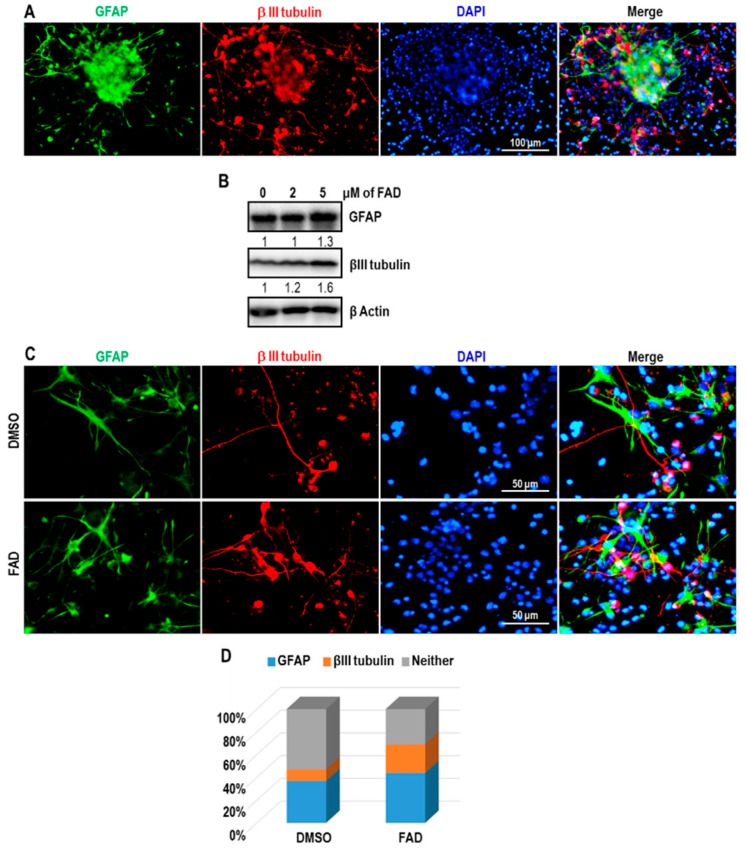
Differentiation induction of NSCs by FAD. (**A**) Immunofluorescence analysis of GFAP (green) and βIII-tubulin (red) expression in NSCs kept under differentiation media for 3 days. Nuclear DAPI (4′,6-diamidino-2-phenylindole) staining is in blue. These are representative results of counting 3 random fields of view. (**B**) Western blot analysis of GFAP and βIII-tubulin in DMSO or FAD-treated cells. β Actin was used as a loading control. The relative band intensities of each proteins are shown below the bands. The intensities of vehicle treated lane were arbitrarily set as 1. (**C**) Immunofluorescence analysis of GFAP (green) and βIII-tubulin (red) expression in NSCs under differentiation condition after treatment of DMSO or FAD for 3 days. (**D**) The quantification of GFAP- and βIII-tubulin-positive cell proportions in DMSO or FAD treated cells. These are representative results of counting 10 random fields of view in each group.

Considering the effect of FAD on the differentiation of glioblastoma stem-like cells, we explored whether FAD could accelerate the differentiation of NSCs. We first checked the differentiation capacity of NSC spheres under NSC differentiation media that contained B-27 supplement and 1% serum without growth factors. As shown in Figure 4A, neuron-specific marker βIII-tubulin and astrocyte marker GFAP were clearly detected upon differentiation induction. Next, we examined the effect of FAD on NSC differentiation. Because 10 μM FAD induced cell death, we treated cells with lower concentrations of FAD (2 μM or 5 μM) and induced differentiation for 3 days. Under enforced differentiation, the levels of both βIII-tubulin and GFAP were upregulated by 5 μM FAD treatment (Figure 4B). To better characterize the induced differentiation by FAD, we performed immunostaining. As a result of counting random fields of view, the percentage of βIII-tubulin-positive neurons was increased approximately 2-fold by 5 μM FAD challenge. Although to a lesser extent than the number of neurons, the number of GFAP-positive astrocytes was also increased by FAD administration (Figure 4C,D). Together, these data suggest that FAD enhances differentiation of NSCs into neurons and astrocytes.

### 2.5. FAD Alters NSC Homeostasis by the Inhibition of Notch Pathway

To figure out the intracellular and molecular mechanism that FAD disrupts the maintenance of NSCs and induces neuronal and glial differentiation, we performed literature analysis: (1) Previously, Yoshida et al. demonstrated that FAD inhibits glycogen synthase kinase-3β [21] that has been repeatedly shown to positively regulate Notch signaling and stability [22,23]. (2) FAD and its derivatives were potent inhibitors of breast cancer resistance protein (ABCG2) [24,25], a well-known target of Notch pathway [26]. (3) FAD suppressed MHC II expression [27] that was known to be induced by Notch signaling [28]. Based on these collective evidence and the importance of Notch pathway in NSC maintenance [29], we hypothesized that FAD would downregulate Notch pathway in NSCs.

**Figure 5:**
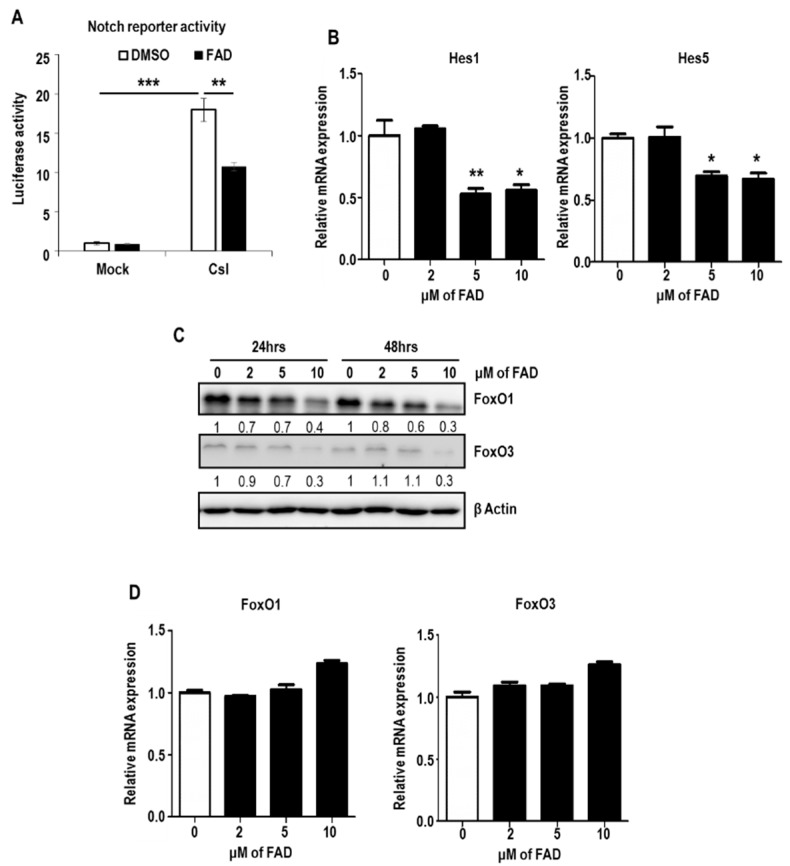
Downregulation of FoxO-Notch axis in NSCs by FAD. (**A**) Csl-induced luciferase reporter activity was measured in DMSO or FAD treated NSCs. Statistical differences were considered significant when ** p < 0.01, and *** p < 0.001. (**B**) The mRNA expression of Hes1 and Hes5 after treatment of DMSO or FAD in NSCs, as measured by real-time RT-PCR analysis. The mRNA level of DMSO-treated cells was set to 1. Representative results from multiple experiments are shown. Statistical significance was determined by unpaired *t*-test. * p < 0.05, and ** p < 0.01. (**C**) Western blot analysis of FoxO1 and FoxO3 in NSCs after treatment of DMSO or FAD for 24 h or 48 h. β Actin was used as a loading control. The relative band intensities of each proteins are shown below the bands. The intensities of vehicle treated lane were arbitrarily set as 1. (**D**) The mRNA expression of FoxO1 and FoxO3 after treatment of DMSO or FAD in NSCs, as measured by real-time RT-PCR analysis. The mRNA level of DMSO-treated cells was set to 1. Representative results from multiple experiments are shown.

To test this hypothesis, Csl-induced Notch reporter activity was measured after treatment of vehicle or FAD. Surprisingly, upon FAD administration, luciferase activity was significantly downregulated (Figure 5A). To confirm the suppression of Notch signaling by FAD, we examined the expression level of Hes1 and Hes5, the major target genes of the Notch pathway. As expected, expressions of both genes were reduced by FAD treatment (Figure 5B). Accumulating evidence suggested that FoxO transcription factor family (especially FoxO1 and FoxO3) is the critical regulator in the maintenance of NSCs [30,31] through the cooperation with Notch pathway [15]. Based on these previous findings, we analyzed the protein levels of FoxO1 and FoxO3. Interestingly, 10 μM FAD treatment caused the obvious reduction of FoxO1 and FoxO3 protein levels (Figure 5C). However, the mRNA expressions of FoxO1 and FoxO3 were not downregulated, meaning that the lowered FoxO1 and FoxO3 protein levels might be due to the inhibition of translation or the acceleration of protein decay, rather than transcriptional decline (Figure 5D). Together, FAD weakens the homeostatic maintenance of normal NSCs by suppressing FoxO-Notch axis. 

## 3. Discussion

As already reported, FAD shows the potent antiproliferative activity, mainly inducing apoptosis and/or cell cycle arrest [32]. Based on these characteristics, FAD has been tested as an antitumor agent. Indeed, FAD was shown to selectively kill colorectal cancer cells and repress tumor growth, without affecting normal colon epithelial cells [13]. The anticancer function of FAD was further demonstrated with breast cancer cells in which FAD contributed to autophagy-dependent tumor cell death [12].

Interestingly, however, FAD did not induce autophagy in our system, suggesting that autophagy works in context or cell type-dependent manner. Indeed, Yordy et al. demonstrated the cell type-specific requirement for autophagy in defense against HSV-1 (DRG neurons vs mitotic cells) [33]. Although we did not find the precise reason why FAD did not induce autophagy in our system, there would be physiological differences between neural stem cells and other cell types, including tumors. In present study, we provide several pieces of evidence suggesting that FAD has the anticancer role by targeting glioblastoma cells, as well as glioblastoma stem-like cells.

Traditional cancer therapies, including chemotherapy and radiation, often failed in tumor treatment due to cancer recurrence. Recently, cancer stem cells have been regarded as the culprits of the tumor relapse, because (1) they generate mature differentiated tumor cells, (2) they have long-term proliferative capacity, and (3) they show high resistance to chemo and radiotherapy. Therefore, the anticancer activity of drug candidates needs to be determined in cancer stem cells, as well as mature tumor cells, and FAD showed the antitumor function in both types of cells in this respect.

Even though FAD had the beneficial effects against cancers, it also showed the cytotoxicity on normal NSCs. Similar to cancer stem cells, NSCs are able to self-renew and proliferate without limit. Because cancer stem cells and NSCs have common regulatory mechanisms to maintain the stemness and cellular homeostasis, such as Notch, Wnt, and Shh signaling, cancer therapy targeting cancer stem cells would be able to show the cytotoxic effect on normal NSCs. In present study, our results suggested that FAD disrupted the homeostasis of both cancer stem cells and NSCs. In addition, FAD reduced the stemness of both cancer stem cells and NSCs, leading to the excessive differentiation. To develop the flawless therapy against cancers, studies to identify precise molecular/cellular mechanisms existing only in mature tumors and tumor stem cells are highly required.

## 4. Methods

### 4.1. Neural Stem Cell Preparation and Differentiation

Primary NSCs were isolated from the brain of mouse embryos and maintained in NSC culture medium supplemented with 20 ng/mL EGF and bFGF. For differentiation induction, NSCs were dissociated into single cells using TrypLE (Life Technologies, Carlsbad, CA, USA), and plated on polyornithine and fibronectin-coated plates in NSC culture medium, including 1% fetal bovine serum (FBS) and B27 Supplements (Life Technologies, Carlsbad, CA, USA) for 4 days.

### 4.2. Cell Culture

The U373 MG human glioblastoma astrocytoma cells were maintained in MEM supplemented with 10% FBS and 1% penicillin–streptomycin in a humidified atmosphere containing 5% CO_2_ at 37 °C. To enrich the cancer stem cell-like populations in glioblastoma cells, U373 cells were adapted in NSC culture medium supplemented with 1% FBS for 6 days and then transferred to the serum-free NSC culture media supplemented with 20 ng/mL EGF and bFGF.

### 4.3. Protein Preparation and Immunoblot Analysis 

Immunoblotting was done as previously described [29]. In brief, cells were disrupted directly with laemmli buffer (60 mM Tris-HCl (pH 6.8), 2% (*w/v*) SDS, 10% (*v/v*) glycerol, 0.02% (*w/v*) bromophenol blue), followed by sonication and heat-denaturation. Immunoblot analyses were fractionated by SDS-PAGE and transferred to a PVDF membrane. After blocking membranes with 5% non-fat dried milk in TBST (10 mM Tris, pH 8.0, 150 mM NaCl, 0.5% Tween 20) for 30 min, the membrane was washed three times (10 min each) with TBST and incubated with antibodies against Bax (1:1000, Bioworld, St. Louis Park, MN USA), γH2AX (1:1000, cusabio, Houston, TX, USA), cleaved Caspase 3 (1:1000, thermo fisher scientific, Waltham, MA, USA), β Actin (1:5000, sigma aldrich, St. Louis, MO, USA), Nestin (1:1000, Novus Biologicals, Littleton, CO, USA), Gfap (1:5000, abcam, Cambridge, UK), phosphor-Histone3 at Ser 10 (1:1000, Cell Signaling, Danvers, MA, USA), phospho-Erk1/2 at Thr202 and Tyr204 (1:1000, thermo fisher scientific), Cyclin D1 (1:1000, Santacruz, Dallas, TX, USA), βIII tubulin (1:1000, abcam), LC3B (1:1000, Novus Biologicals), FoxO1 (1:1000, thermo fisher scientific), and FoxO3 (1:1000, thermo fisher scientific) overnight at 4 °C. Next day, membranes were washed three times (10 min each) with TBST and incubated with horseradish peroxidase-conjugated anti-mouse (1:10,000, Bethyl Laboratories, Montgomery, TX, USA) or anti-rabbit antibodies (1:5000, Bethyl Laboratories) for 1 h. Membranes were washed three times (10 min each) with TBST and signals were detected with Clarity^TM^ Western ECL Substrate (bio-rad, Hercules, CA, USA).

### 4.4. Immunofluorescence

For immunofluorescence, cells were fixed with 4% paraformaldehyde and permeabilized with 0.2% Triton X-100/PBS for 15 min each at room temperature. After blocking samples with 2% BSA/PBS for 30 min, cells were subjected to immunofluorescence staining with anti-Gfap (1:1000, abcam), anti-Nestin (1:100, Novus Biologicals), anti-phosphor-Histone3 at Ser 10 (1:200, Cell Signaling), and anti-βIII tubulin (1:200, abcam) primary antibodies overnight at 4 °C. Next day, cells were washed with cold PBS and incubated with Flamma®552- or Flamma®488- conjugated goat anti-rabbit IgG (bioacts) or goat anti-mouse IgG (bioacts, Incheon, Korea) for 30 min at room temperature. Fluorescence signals were visualized with EVOS FL Auto Imaging System (thermo fisher scientific).

### 4.5. Quantitative Real-Time RT-PCR

Total RNA was isolated using RNA Purification Kit (thermo fisher scientific). 200 ng of total RNA was treated with RNase-free DNase (sigma aldrich) for 15 min. After inactivation of DNase with EDTA and heating, RNA was reverse transcribed using First Strand cDNA Synthesis Kit (thermo fisher scientific) according to the manufacturer’s instructions. Quantitative RT-PCR was performed on cDNA samples using the Power SYBR Green Master mix and was performed the qPCR on the Mic qPCR Cycler (bio molecular systems, Upper Coomera, Australia). The relative mRNA level was calculated as values of 2^(Ct(β-actin)−Ct(gene of interest)). For data presentation, the mRNA level in control cell was set to 1. The sequences of the forward and reverse primers are as follows: β actin (mouse), 5′-GGCTGTATTCCCCTCCATCG-3′ and 5′-CCAGTTGGTAACAATGCCATGT-3′; Hes1 (mouse), 5′-CCAGCCAGTGTCAACACGA-3′ and 5′-AATGCCGGGAGCTATCTTTCT-3′; Hes5 (mouse), 5′-GCAGCATAGAGCAGCTGAAG-3′ and 5′-AGGCTTTGCTGTGTTTCAGG-3′; FoxO1 (mouse), 5′-TTCAATTCGCCACAATCTGTCC-3′ and 5′-GGGTGATTTTCCGCTCTTGC-3′; FoxO3 (mouse), 5′-AGCAAGCCGTGTACTGTGG-3′ and 5′-GAGCGCGATGTTATCCAGC-3′; β actin (human), 5′-CATGTACGTTGCTATCCAGGC-3′ and 5′-CTCCTTAATGTCACGCACGAT-3′; Bax (human), 5′-CCCTTTTGCTTCAGGGTTTC-3′ and 5′-ATCCTCTGCAGCTCCATGTT-3′; Bad (human), 5′-CGGAGGATGAGTGACGAGTT-3′ and 5′-ACTTCCGCCCATATTCAAGA-3′; Bak (human), 5′-TCTGGCCCTACACGTCTACC-3′ and 5′-ACAGAACCACACCCAGAACC-3′; p21 (human), 5′-GACACCACTGGAGGGTGACT-3′ and 5′-CAGGTCCACATGGTCTTCCT-3′.

### 4.6. Transfection and Reporter Assays

For Notch reporter plasmid transfection, NSCs were dissociated and plated in NSC culture medium, including EGF and bFGF. After 24 h incubation, firefly luciferase mock vector or 4XCSL-luciferase reporter plasmid (a gift from Raphael Kopan, Addgene plasmid # 41726) [34] was co-transfected with a TK-renilla plasmid using Lipofectamine 3000 (Life Technologies) according to the manufacturer’s instruction. At 12 h after transfection, cells were treated with DMSO or 10 μM FAD for additional 24 h. Harvested cells were subjected to the luciferase activity measurement. The relative luciferase activity was determined as the ratio of firefly to Renilla activity. For data presentation, the luciferase activity of pGL3 mock-negative control vector was set to 1. 

### 4.7. Statistical Analysis

The unpaired two-tailed Student’s *t*-test was used for experiments comparing two sets of data unless noted. All results are expressed as mean ± s.e.m. GraphPad Prism software (version 6, San Diego, CA, USA) was used for all statistical analysis. Differences were considered significant when * p < 0.05, ** p < 0.01, and *** p < 0.001.

## Supplementary Materials

The following are available online at http://www.mdpi.com/1422-0067/19/11/3432/s1.
