# Supplementary material for: The Antitumor Natural Compound Falcarindiol Disrupts Neural Stem Cell Homeostasis by Suppressing Notch Pathway
Source: Int J Mol Sci. 2018 Nov 1;19(11):3432. doi: 10.3390/ijms19113432 (PMC6274977; doi:10.3390/ijms19113432)
Supplement: Supplementary file 1 [file ijms-19-03432-s001.pdf]

Supplementary Figure 1

A

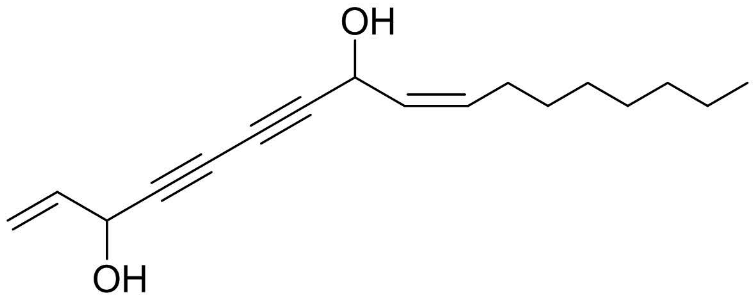

B

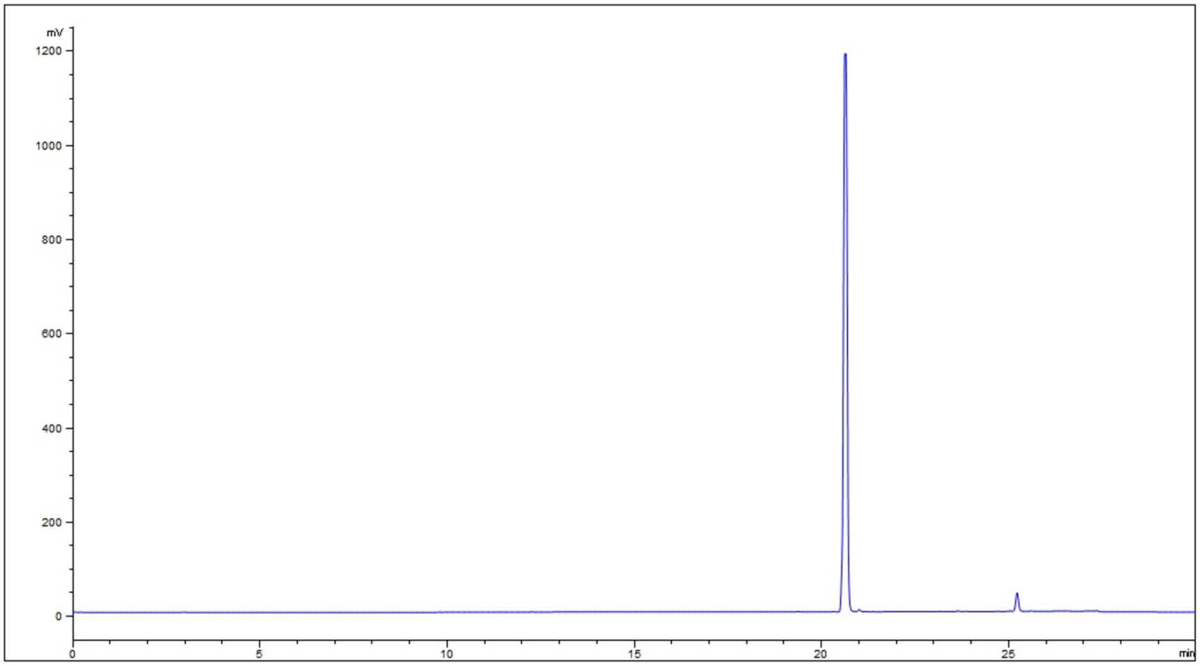

Supplementary Figure 1. Isolation of falcarindiol.

(A) Chemical structure of falcarindiol isolated from roots of *S. divaricate*. (B) HPLC chromatogram of falcarindiol isolated from roots of *S. divaricate*.

Supplementary Figure 2

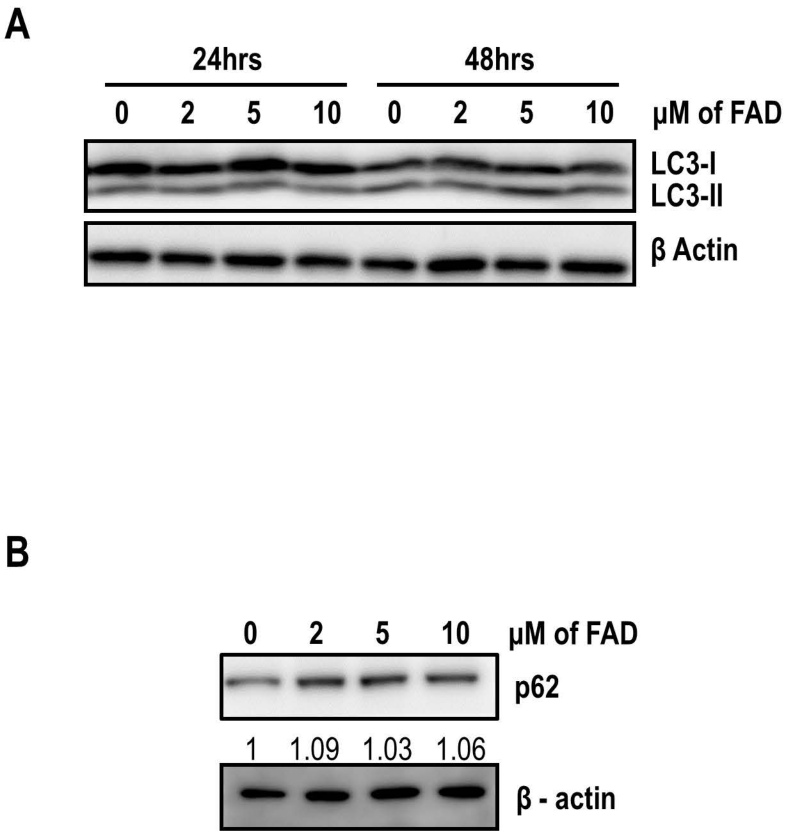

Supplementary Figure 2. FAD does not induce autophagy.

(A) Western blot analysis of LC3 proteins in vehicle (0) or FAD (2, 5, 10 µM) treated NSCs. Cells were incubated with vehicle or FAD for the indicated time. β Actin was used as a loading control. (B) Western blot analysis of p62 proteins after FAD treatment. Vehicle (0) or FAD (2, 5, 10 µM) was treated for 48hrs. β Actin was used as a loading control. The relative band intensities of p62 proteins are shown below the bands. The intensity of vehicle treated lane was arbitrarily set as 1.
